# Strengthening Policy Relevance of Wastewater-Based Surveillance for Antimicrobial Resistance

**DOI:** 10.1021/acs.est.4c09663

**Published:** 2025-01-28

**Authors:** Sheena Conforti, Amy Pruden, Nicole Acosta, Christopher Anderson, Helmut Buergmann, Juliana Calabria De Araujo, Judith R. Cristobal, Barbara Drigo, Claire Ellison, Zanah Francis, Dominic Frigon, Markus Gaenzle, Julia Vierheilig, Timothy R. Julian, Uli Klümper, Liping Ma, Chand Mangat, Maya Nadimpalli, Manami Nakashita, Gilbert Osena, Sasikaladevi Rathinavelu, Richard Reid-Smith, Michael Saldana, Heike Schmitt, Shuxian Li, Andrew C. Singer, Tam T. Tran, Kadir Yanac, Gustavo Ybazeta, Monika Harnisz

**Affiliations:** † Eawag, Swiss Federal Institute of Aquatic Science and Technology, Dübendorf 8600, Switzerland; b Civil and Environmental Engineering, Virginia Polytechnic Institute and State University, 418 Durham Hall, Blacksburg, Virginia 24061, United States; c University of Calgary, Cumming School of Medicine, Calgary, AB T2N 1N4, Canada; d West Virginia University, Morgantown, West Virginia 26506-6201, United States; e Eawag, Swiss Federal Institute of Aquatic Science and Technology, Kastenienbaum 6047, Switzerland; f Federal University of Minas Gerais, Belo Horizonte, MG 31270-901, Brazil; g Department of Chemistry, University at Buffalo - The State University of New York, 633 Natural Science Complex, Buffalo, New York 14260, United States; h University of South Australia, Adelaide, SA 5001, Australia; i Queen’s University, Beaty Water Research Center, Kingston, ON K7L 3N6, Canada; j U.S. Department of Health and Human Services, Washington, D.C. 20201-0004, United States; k McGill University, Civil Engineering and Applied Mechanics, 817 Sherbrooke Street West, Montreal, QC H3A 0C3, Canada; l University of Alberta, Edmonton, AB T6G 2R3, Canada; m TU Wien, Institute of Water Quality and Resource Management, ICC Water & Health, 1040 Wien, Austria; n Institute for Hydrobiology, TU Dresden, Dresden 01062, Germany; o East China Normal University, Dongchuan Road 500, Shanghai 200241, China; p Public Health Agency of Canada, Wastewater Surveillance Unit, National Microbiology Laboratory, Winnipeg, MB R3E 3R2, Canada; q Gangarosa Department of Environmental Health, Emory University, Atlanta, Georgia 30322, United States; r National Institute of Infectious Diseases, Shinjuku-ku, Tokyo 162-8640, Japan; s University of Gothenburg, Goteborg, Västra Götaland 405 30, Sweden; t Public Health Agency of Canada Foodborne, Waterborne and Zoonotic Infections Division, Guelph, ON N1G 5B2, Canada; u Sonny Astani Civil and Environmental Engineering, University of Southern California, 920 Downey Way, BHE 201, Los Angeles, California 90089-0001, United States; v National Institute for Public Health and the Environment, Bilthoven 3720 BA, The Netherlands; ∇ Delft University of Technology, Delft, Zuid-Holland 2600 AA, Netherlands; w Department of Civil Engineering, The University of Hong Kong, Hong Kong 999077, China; x Centre for Ecology & Hydrology, Mansfield Road, Oxford OX1 3SR, United Kingdom; y NORCE Norwegian Research Centre AS, Tromso, Troms og Finnmark 9019, Norway; z University of Manitoba, Department of Civil Engineering, Winnipeg, MB CR3T 5V6, Canada; Ω Health Sciences North Research Institute, Sudbury, ON P3E 2H2, Canada; Σ University of Warmia and Mazury in Olsztyn, Department of Water Protection Engineering and Environmental Microbiology, Prawochenskiego 1, Olsztyn 10-790, Poland

**Keywords:** antimicrobial resistance, wastewater-based surveillance, public health, policy integration, One Health, epidemiology

Antimicrobial resistance (AMR) is among the top 10 public health threats, with nearly 5 million deaths in 2019 linked to AMR-related bacterial infections.^1^ A One Health approach is needed to combat AMR.

Healthcare-based surveillance (HBS) of AMR provides incomplete information about the scope of the AMR threat. HBS screens only patients seeking medical attention, lacking community-level representativeness, and suffers from underreporting.^2^ Consequently, researchers are turning to wastewater-based surveillance (WBS) to complement HBS.^3^ WBS can provide information about AMR circulating within communities and hospitals, offering a comprehensive understanding of AMR prevalence. However, the surveillance targets and data obtained from WBS are distinct from those derived from HBS, creating uncertainty regarding their utility to the public health sector and ability to yield policy relevant information. In May 2024, participants in a workshop during the 7^th^ Environmental Dimension of Antimicrobial Resistance (EDAR7) conference (Montréal, Canada) sought to answer four questions aimed at advancing the policy relevance of AMR data generated by WBS.

## What Public Health Relevant Indicators Are Currently Used to Drive Antimicrobial Stewardship Policy?

**Figure 1:**
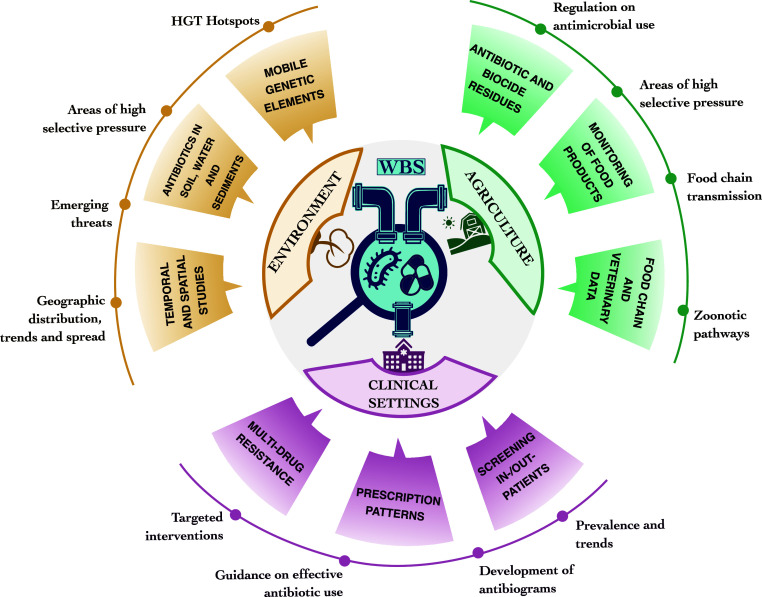
Integration of data across One Health sectors to inform AMR policy and the potential role of WBS. Clinical settings, environmental studies, and agricultural surveillance can provide indicators used for antimicrobial stewardship and antimicrobial resistance (AMR) management. Indicators include prevalence of AMR and prescription patterns in clinical settings, geographic distribution and emerging threats in the environment to identify horizontal gene transfer (HGT) hot spots, and antibiotic/biocide residues and zoonotic pathways in agriculture. Wastewater-based surveillance (WBS) programs can be designed to integrate within and across all sectors.

There is a pressing need to integrate available information across One Health sectors (human health, agriculture, and environment) to inform policy and practice aimed at mitigating AMR (Figure 1). HBS aims to guide antibiotic prescriptions by generating antibiograms and provides data on AMR prevalence and trends by prescreening inpatients for carriage, assessing resistance of pathogens responsible for infections, and tracking in- and out-patient antibiotic prescription patterns. Tracking trends of multidrug-resistant (MDR) organisms in healthcare facilities helps to identify units experiencing high rates of nosocomial infections and informs the selection of appropriate treatment options. Well-established surveillance programs can result in public health reports used to define strategies to regulate antibiotic stewardship and to monitor and evaluate interventions.

Surveillance in agriculture tends to be more focused on antibiotic use than tracking resistant infections, although some monitoring programs track resistant isolates in meat, produce, and other food products. Measuring antibiotic consumption in animals and crops can help identify hot spots for selective pressure and potential for AMR to spread. Monitoring residues in food of animal origin and biocides in vegetables can also contribute. Surveillance of AMR in livestock, wild animals, and the food chain can inform transmission pathways between animals and humans, providing insights into interventions to curb foodborne and zoonotic spread. Agricultural surveillance supports the establishment and enforcement of antibiotic stewardship in animals and plants, including antibiotic use regulations.

There is increasing recognition of the environmental dimension of One Health approaches to inform AMR policy, but environmental surveillance programs, including WBS, are still limited. Environmental indicators of AMR, such as the presence and concentration of antibiotic resistance genes (ARGs) and resistant organisms in wastewater effluents, rivers, lakes, air, and soils, are primarily derived from spatial and longitudinal studies. These studies identify hot spots of resistance, guide environmental regulations like wastewater management policies, and inform agricultural practices to reduce antibiotic runoff. Environmental surveillance is also uniquely positioned for identifying emerging threats, including new ARGs, mobile genetic elements (MGEs), biocidal resistance genes, and resistant organisms. Monitoring targeted sources, including human, industrial, and agricultural wastewaters, has identified hot spots of resistance and selective pressure. The study of MGEs, in particular, offers insights into the mobility of ARGs and the potential acquisition of new resistance mechanisms in pathogens.

## What Public Health Relevant Targets and Data Can Be Derived from Wastewater through Monitoring Programs?

WBS can include monitoring of antimicrobials, resistant organisms, ARGs, and MGEs in human, industrial, or agricultural/food production wastewaters, with monitoring locations selected to integrate across specific sources of interest. However, there is a need to better strategize and coordinate WBS of AMR in a manner that focuses on targets and data that are very likely to provide actionable information. One strategy could be prioritizing low-prevalence resistant bacteria of high clinical relevance, such as carbapenemase-producing Enterobacterales, vancomycin-resistant *Enterococcus* spp., or other bacteria of the World Health Organization Bacterial Priority Pathogen List.^4^ An increase in the level of resistant organisms in wastewater can indicate rising community-level carriage, potential outbreak risks, or intervention failures. WBS may also help determine if outbreaks have ended or if asymptomatic cases persist in the community. However, it is important to be aware of population-scale detection limits and to determine the necessary temporal resolution (e.g., weekly monitoring) to achieve the monitoring goal. In contrast, monitoring pathogens that are already widespread does not necessarily add significant value to inform public health actions.

Metagenomic approaches, i.e., sequencing of DNA across microbial populations encountered in wastewater, can offer a comprehensive view of ARGs and MGEs circulating within the corresponding population. Because metagenomics is a nontargeted approach, this perspective could identify emerging ARGs or provide an early warning regarding acquisition of ARGs by pathogens of concern in a community. For example, early detection of the mcr-1 gene conferring resistance to colistin through metagenomics led to the implementation of stricter colistin stewardship and monitoring in high-risk areas, such as units with high rates of MDR.^5^

WBS can also target antimicrobials, thus filling knowledge gaps regarding the patterns and prevalence of the use of antimicrobials and other pharmaceuticals. Efforts are needed to improve reporting of antimicrobial use data. Where data are available, they tend to be highly aggregated and costly and with low spatial and temporal resolution. However, antibiotic testing does require sophisticated instrumentation and expertise and works best for antibiotics, such as macrolides and fluoroquinolones, that persist longer in wastewater environments. Fast-degrading antibiotics such as β-lactams might still be detected in the outflow from hospitals with short retention times.

A general advantage of WBS is the ability to capture longitudinal and spatial trends across populations and sources of interest. Notably, different sanitation infrastructures and spatial scales of WBS provide distinct opportunities for measurement and interpretation. For example, in hospital wastewater, the indicators reflect carriage of resistant organisms or antibiotic usage within a specific facility. In municipal wastewater, the indicators reflect trends of resistance or antibiotic consumption within the community. Importantly, most of the world is served by nonsewered sanitation, particularly in low- and middle-income countries; surveillance in these settings might focus on tracking emergence and estimating prevalence in specific community settings (e.g., schools, universities, and hospitals). WBS can help to fill critical knowledge gaps in HBS, particularly in countries lacking comprehensive diagnostic capabilities.

## What Information, Resources, and Contextualization Are Needed to Align Public Health Indicators Derived from Wastewater with Other Public Health Indicators to Better Inform Our Epidemiological Understanding of AMR?

A key consensus of the workshop was the need to integrate WBS data with HBS to better inform public health strategies.

Information needed includes data on AMR prevalence from clinics, meaning the pathogens encountered in the population and corresponding rates of resistance to specific antibiotics obtained through HBS. Such monitoring can reveal clinically relevant targets for WBS and allow the establishment of standard methodologies for consistent data collection and interpretation. Whole genome sequencing of human and animal clinical strains can provide information needed to calibrate WBS data and track persistent pathogens and ARGs of concern in wastewater, potentially indicating ongoing transmission. Information about antibiotic usage in humans, animals, and plants, prescription practices, rates of antibiotic degradation in wastewater, flow data, and transport in sewage systems will help better align WBS and healthcare sector AMR indicators.

Resources necessary for advancing WBS of AMR include institutional, financial, and human capital investments. These can support the design, implementation, and continuity of a monitoring plan to yield comprehensive and longitudinal data collection needed to infer AMR dynamics within the community. Initial costs for setting up laboratories, building infrastructure, and establishing workflows among stakeholders such as those who operate wastewater facilities and other monitoring locations are necessary to centralize analyses and build capacity. The investments made in infrastructure and organization for COVID-19 surveillance, and increasingly other pathogens, provide an opportunity to leverage existing resources for AMR monitoring. Establishing publicly accessible databases to collect, visualize, and analyze data from both wastewater and clinical surveillance will enhance collaboration among clinicians, policy makers, researchers, and other stakeholders.

WBS indicators for AMR should be contextualized with respect to clinical and agricultural/food sector surveillance through strong collaborations among researchers, clinicians, and communities. While WBS alone may not always generate information about specific targets of interest, it can identify broader trends and emerging hot spots and inform public health strategies like early warnings and antimicrobial stewardship efforts. Notably, transitioning from WBS to wastewater-based epidemiology for AMR poses significant challenges, for example, in predicting the prevalence of AMR within the population. Complications include the dynamics and complexity of pathogen shedding rates and antibiotic resistance mechanisms, and the growth, fate, and transport processes in sewer networks. One key issue is the potential proliferation of resistant organisms within the sewer network, both in the wastewater and in biofilm, which can decouple wastewater-based quantitative estimates from inferences about AMR epidemiology. Indicators from WBS could be developed to help inform progress toward the Sustainable Development Goals or otherwise provide insight into key socioeconomic factors driving overall trends. Geographical and mobility patterns within sewersheds, and connections between industries and hospitals, should be considered to calibrate wastewater indicators and discern community-sourced data from other origins. Research on the fate of resistant bacteria in wastewater systems, along with cohort studies on resistant bacteria in human carriers, may help improve our understanding and interpretation of WBS-derived data.

## How Can the Information Derived from WBS of AMR Contribute to the Formulation of Effective Public Health Policies or Interventions?

WBS offers population-integrated data at comparatively low cost and effort relative to monitoring individuals within a population. It provides broader views on population prevalence, independent of screening effort, participation rates, and the likelihood of reporting to health services. Additionally, it enables a comprehensive overview of the microbial genomes circulating in the environment and provides space- and time-resolved data that can be scaled to various needs. As critiqued in the recent 2024 NASEM report, we acknowledge the limitations of WBS for ARG-focused monitoring at the community level, which can be complicated by ARGs from non-human sources and the amplification of ARGs between the human source and the wastewater treatment plant.^6^ However, it is important to recognize that WBS of AMR could provide much broader value beyond serving as an early warning system, especially in terms of evaluating long-term trends and effects of policy interventions on shaping these trends. We highlight alternative use cases that are of particular value for aligning WBS data with actionable public health objectives and HBS, such as detection of the emergence of novel resistance genes, or use of culture- and molecular-based methods to track long-term changes in community prevalence rates.^7^

Integrating WBS data with existing surveillance methods (Figure 1) is a promising approach to enhance AMR understanding by correlating wastewater findings with clinical data, making policies actionable. WBS data can expand and provide greater resolution to traditional clinical antibiograms while also filling diagnostic gaps and better optimizing the selection of antibiotic treatments in regions with limited spatial and longitudinal AMR data.

Public access and education, e.g., via media outlets, can increase AMR awareness, thereby enhancing public support and compliance with AMR policies. To inform effective public health interventions from WBS, it is necessary to have clear objectives and collaborate closely with stakeholders across One Health sectors, which can facilitate the implementation of targeted and efficient measures aimed at limiting the evolution and transmission of antibiotic-resistant pathogens.
